# Perceived causes of differential attainment in UK postgraduate medical training: a national qualitative study

**DOI:** 10.1136/bmjopen-2016-013429

**Published:** 2016-11-25

**Authors:** Katherine Woolf, Antonia Rich, Rowena Viney, Sarah Needleman, Ann Griffin

**Affiliations:** Research Department of Medical Education, UCL Medical School, London, UK

**Keywords:** EDUCATION & TRAINING (see Medical Education & Training), QUALITATIVE RESEARCH, postgraduate education, ethnicity, diversity, international medical graduate

## Abstract

**Objectives:**

Explore trainee doctors’ experiences of postgraduate training and perceptions of fairness in relation to ethnicity and country of primary medical qualification.

**Design:**

Qualitative semistructured focus group and interview study.

**Setting:**

Postgraduate training in England (London, Yorkshire and Humber, Kent Surrey and Sussex) and Wales.

**Participants:**

137 participants (96 trainees, 41 trainers) were purposively sampled from a framework comprising: doctors from all stages of training in general practice, medicine, obstetrics and gynaecology, psychiatry, radiology, surgery or foundation, in 4 geographical areas, from white and black and minority ethnic (BME) backgrounds, who qualified in the UK and abroad.

**Results:**

Most trainees described difficult experiences, but BME UK graduates (UKGs) and international medical graduates (IMGs) could face additional difficulties that affected their learning and performance. Relationships with senior doctors were crucial to learning but bias was perceived to make these relationships more problematic for BME UKGs and IMGs. IMGs also had to deal with cultural differences and lack of trust from seniors, often looking to IMG peers for support instead. Workplace-based assessment and recruitment were considered vulnerable to bias whereas examinations were typically considered more rigorous. In a system where success in recruitment and assessments determines where in the country you can get a job, and where work–life balance is often poor, UK BME and international graduates in our sample were more likely to face separation from family and support outside of work, and reported more stress, anxiety or burnout that hindered their learning and performance. A culture in which difficulties are a sign of weakness made seeking support and additional training stigmatising.

**Conclusions:**

BME UKGs and IMGs can face additional difficulties in training which may impede learning and performance. Non-stigmatising interventions should focus on trainee–trainer relationships at work and organisational changes to improve trainees’ ability to seek social support outside work.

**Strengths and limitations of this study**

- This is the first study to explore how ethnicity affects UK-qualified doctors' experiences of postgraduate medical training. It therefore provides valuable insights into the causes of black and minority ethnic UK graduates' underperformance in postgraduate assessments and recruitment, and provides a basis on which interventions to reduce differential attainment can be developed and evaluated.
- The study has a large and diverse sample, comprising trainees from white and black and minority ethnic backgrounds, UK and international graduates, across six medical specialities, four geographical areas in England and Wales, and all training grades. It also includes trainers, programme directors and postgraduate deans. This allows in-depth analysis of the issues from a range of perspectives.
- Selection bias is a possibility, although the data showed a wide variety of views. Related to that, data were collected in November and December 2015 during the junior doctor contract dispute which may have led to trainees vocalising greater discontent with their training than usual, although the findings did not suggest doctors from dissimilar backgrounds perceived the new contract differently.
- Low recruitment from some specialties, for example, radiology, did not permit comparison of potential differences between specialties.

## Introduction

International medical graduates (IMGs) are more likely to fail postgraduate assessments and have poorer outcomes in recruitment in the UK, USA, Canada and Australia.^1–6^ Doctors from black and minority ethnic (BME) groups also have poorer academic and recruitment outcomes compared with white doctors in the UK, USA, the Netherlands and Australia^1^ ^7–9^ and in higher education (HE) more generally.^10–12^ These group differences are known as differential attainment and pose a significant problem for the medical profession. Healthcare provision relies on IMGs,^1^ ^13^ and medicine is a very popular choice for BME students.^14^ In the UK, public authorities such as universities, Royal Colleges and the National Health Service (NHS) have a legal duty to address differences between groups with and without the protected characteristic of ‘race’ (which covers ‘race, colour, and nationality (including citizenship) ethnic or national origins’).^15^ In 2014, the Membership of the Royal College of General Practitioners (MRCGP) examination and the General Medical Council (GMC) were brought to judicial review over differential attainment^15^ ^16^ raising the profile of the problem.

IMGs are known to face challenges including adapting to a new culture and style of teaching and learning, new language, change in hierarchy, discrimination, and the psychological impacts of migration.^9^ ^17–19^ Much less is known about the causes of the ethnic attainment gap among UK graduates (UKGs), and it is unclear whether IMGs and BME UKGs have experiences in common. A 2015 GMC-commissioned rapid review of the literature^20^ highlighted a lack of consensus and research about the causes of the ethnic attainment gap in UKGs. There is however general agreement that examiner bias or overt discrimination is unlikely to be the sole cause in examinations in medicine because differential attainment is seen in written machine-marked multiple choice examinations,^21^ and research into two postgraduate clinical examinations found no evidence of bias.^22^ ^23^ This has shifted the focus of differential attainment research onto understanding experiences and opportunities.

This shift is reflected in a recent Higher Education Funding Council England (HEFCE)-commissioned report into causes of ethnic differences in the UK HE.^24^ Four categories of explanatory factors were identified: (1) students' experiences of HE learning, teaching and assessment; (2) relationships that underpin students' experiences of HE; (3) psychosocial and identity factors; and (4) cultural and social capital factors. This report was important because it moves understanding on from the ‘deficit model’ whereby differences are attributed to student deficits such as poorer previous attainment, lower motivation, poorer preparation for university, none of which can fully explain ethnic differences.^25^ ^26^ The current study was part of a GMC-funded workstream on differential attainment, and aimed to explore trainee doctors' experiences of postgraduate medical training and their perceptions of its fairness, using the HEFCE framework as a guide to identify causes of differential attainment by ethnicity and country of qualification (UK vs non-UK).

## Methods

### Design

We took a qualitative approach to gain understanding of trainees' lived experiences of training and progression.^27^ Data were gathered in focus groups and one-to-one interviews in person and over the phone, using a semistructured interview guide (see online supplementary appendix), which was piloted on two junior doctors. Trainee experiences were contextualised by views of trainers, programme directors and postgraduate deans. All participants received a certificate of participation and focus group members received refreshments.

10.1136/bmjopen-2016-013429.supp1 supplementary data

### Participant sampling framework and recruitment

In the UK medical training, an undergraduate medical course is followed by postgraduate training comprising two foundation years and then specialty training (ST). In England, postgraduate training is organised into geographical areas administered by Health Education England (HEE) Local Education and Training Boards (LETBs); in Wales, it is organised by the Welsh Deanery.

We sampled across five LETBs in England (Kent Sussex and Surrey (KSS), North Central and East London, North West London, South London, Yorkshire and Humber), the Welsh Deanery and the corresponding foundation schools, all chosen because they have varying proportions of IMGs/UKGs, and varying average postgraduate examination performance. Our sampling frame included trainees from four ethnic/country groups (BME UKG, white UKG, BME IMG and white IMG), from six specialties with differing competition ratios and proportions of IMGs/UKGs and white/BME doctors (medicine, surgery, psychiatry, general practice, clinical radiology, obstetrics and gynaecology) plus foundation training, and across training (foundation, ST years 1–3, and 4+) as well as doctors who had failed to progress in their training, or who had completed their training within the last year. Participants were eligible if they were currently in training, had recently completed training, or had failed to progress, or were trainers in one of the specialties or foundation in one of the geographic regions.

Participants were recruited in three main ways: (1) all participating LETBs/deaneries and foundation schools emailed invitations to all their trainees and trainers; (2) we invited people attending events (three general practitioner (GP) events in KSS, one radiology event in London, one orthopaedic surgery event in London, one mixed specialty event in London) to take part either immediately after the event or to express interest in taking part at a later date; and (3) advertised in the Royal College of Physicians President's newsletter. Aside from those who took part immediately after an event, potential participants were asked to contact the research team if they were interested in taking part, and those who did were sent an online survey asking them their gender, ethnicity, country of primary medical qualification (medical school), stage of training, specialty (if relevant) and whether they were willing to participate in a focus group, interview or either.

We organised four trainee focus groups in different towns in Yorkshire and Humber, three in London, one in KSS and one in Wales. Venues were local universities or hospitals. Eligible participants who responded to the survey were invited to attend a local focus group or to be interviewed. Owing to high interest we were unable to interview everyone and chose participants deliberately to populate our sampling frame.

### Analysis

Data were analysed using QSR NVivo V.10 and following Braun and Clark.^27^ KW (academic psychologist), AR (health psychologist) and RV (linguist) read through all transcripts individually and identified themes that emerged from the data, using Mountford-Zimdars and colleagues' analytic framework as a guide. Specifically, we looked for evidence that Mountford-Zimdars' four main themes (curricula and learning, psychosocial and identity factors, relationships, and social, cultural, and financial capital) were present and identified the codes that made up those themes, and also allowed any additional codes and themes to emerge from the data. We then met to discuss our findings, and agree a first coding framework. KW, AR and RV coded three transcripts individually using the agreed coding framework, which we then refined after further discussion. RV then coded the entire data set using the final framework. Subsets of the data were second-coded by each member of the research team (including SN, a clinical oncology trainee and clinical teaching fellow, and AG, a GP and medical educator); consistency was ensured by discussing the framework with all team members and agreeing descriptors for each code before coding. Differences between RV's and the other team members' coding were resolved through discussion, with RV making the necessary adjustments to the final coded version of the data set. This final coded data set was used to write up the findings.

Participants gave informed consent before taking part.

## Results

### Participants

**Figure 1:**
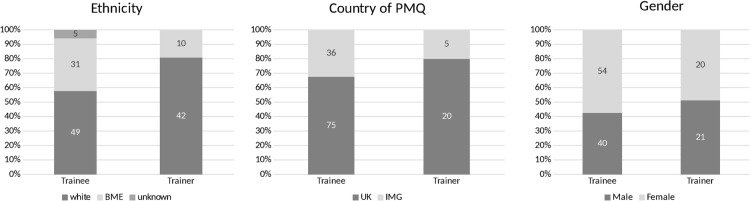
Participant demographics. BME, black and minority ethnic; IMG, international medical graduate; PMQ, primary medical qualification.

Three hundred and ninety-two trainees and trainers expressed interest and 137 (96 trainees including 1 post-completion of training and 1 who failed to progress; 41 trainers) participated. Data were gathered in October, November and December 2015 in 13 focus groups and 35 one-to-one interviews with trainees, and 3 focus groups (all GPs at KSS) and 14 one-to-one interviews with trainers. Participant demographics are shown in figure 1.

### Perceived causes of differential attainment

**Figure 2:**
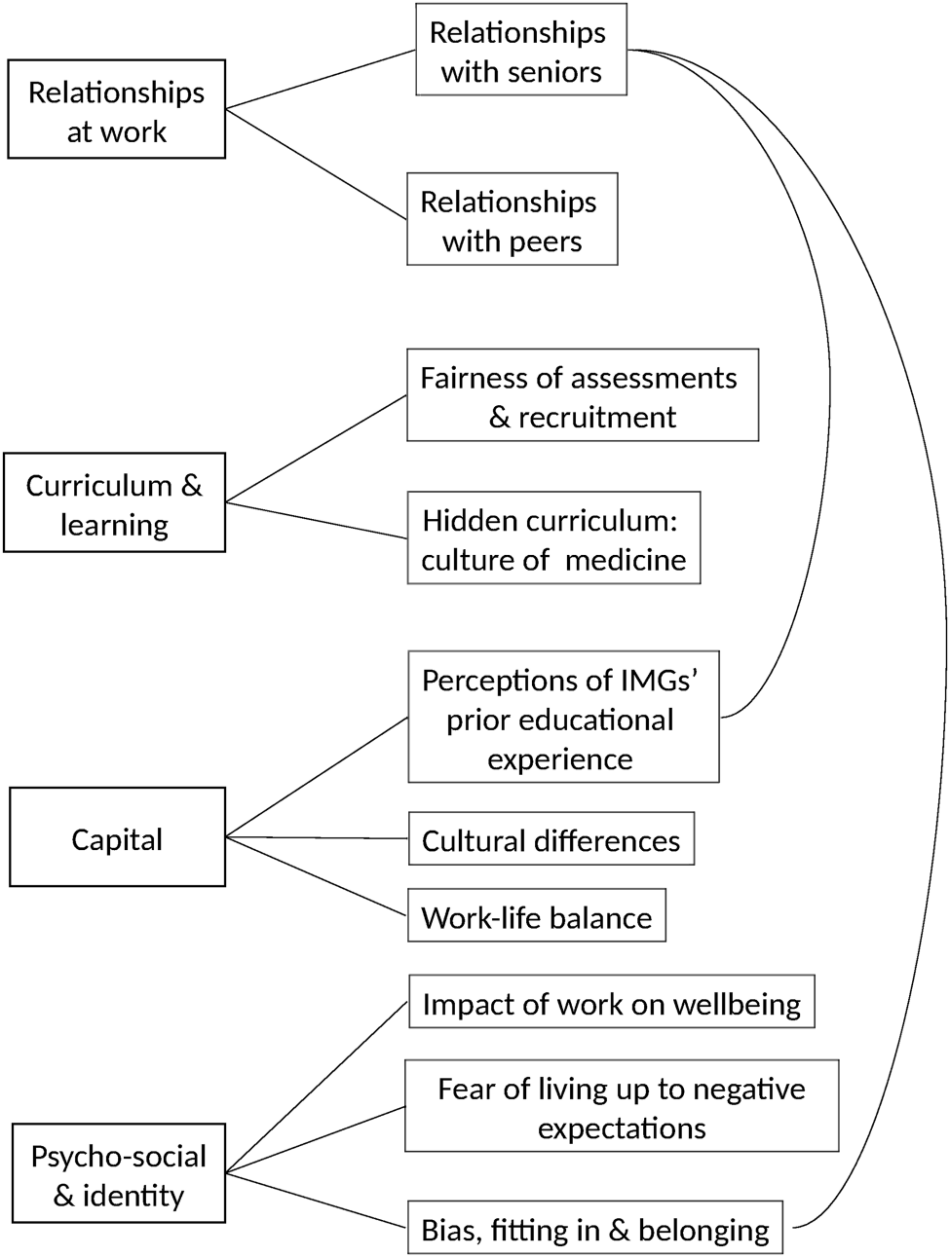
The main themes (left) and subthemes (right) describing the difficulties faced by BME UKGs and/or IMGs that could cause differential attainment. The subtheme ‘relationships with seniors’ was linked to two subthemes within the main theme ‘capital’ as illustrated by the curved lines. BME, black and minority ethnic; IMG, international medical graduate; UKG, UK graduate.

Most trainees had experienced difficulties during training but several themes and subthemes were identified that described how additional difficulties faced by BME UKGs and/or IMGs were perceived to cause differential attainment—see figure 2.

#### Relationships with senior doctors

Relationships with senior doctors were perceived as crucial to learning. At best seniors gave trainees confidence by providing them with opportunities to take responsibility for patients, giving constructive feedback and reassuring about problems including examination failure. Building confidence was especially important in extremely busy, understaffed or disorganised environments in which trainees had little choice but to take responsibility. When seniors did not believe in trainees' abilities, were bullying, blamed trainees or were perceived not to care, trainees' confidence could be damaged for months and the lack of confidence could follow them into subsequent jobs. The same trainee could be treated positively by one senior and negatively by another, hugely affecting confidence and success.

I had a six month experience with a boss where I learned how to be resilient, and I learned how to take the knocks, but I didn't learn a great deal […]. Whatever I could do beforehand was questioned. […] I sort of just kept my head above the water. […] After that [I] spent about a year basically getting my confidence back.

 (Asian other UKG male ST4+ surgery)

From day 1 it was criticism. I had a college tutor walk up to me once and told me “Anaesthetics is not for everybody, you can get a job as a resident medical officer”. So that stayed at the back of my mind for quite another 5, 6 months while I was there. It was getting unhealthy for me, I was getting a lot of psychological emotional stress, so I decided before I leave anaesthetics let me see if other hospitals are like that. […] And within the first month of me working [at another hospital] […] the college tutor there, called me and said “you seem to be not confident about anything, and we've had someone assess you, she thinks your skills are good […] just relax and pay attention to the work”. [laughs] […] I decided to stay on with that encouragement, with a little bit of effort, and I went on to finish my final anaesthesia fellowship.

(Black IMG male ST1–3 GP)

BME UKGs and IMGs in our sample were less likely to report support from seniors in pressurised situations and more likely to say seniors did not believe in them. There were several potential reasons for this, as described below.

##### Cultural differences

Cultural differences could impede good educational relationships for IMGs. It was generally agreed that IMGs who found it difficult to adapt to UK patient-centred care and who—even if they spoke English as a first language—struggled with colloquialisms, would struggle with colleagues. UKGs felt IMGs would struggle with patients too and trainers including an IMG reported difficulties teaching trainees who behaved culturally inappropriately. IMG trainees felt cultural difficulties affected their relationships with colleagues more than with patients. They described how difficult it could be to learn new cultural norms especially if they had to ‘unlearn’ previously acquired knowledge or if UK norms were very different.

I've been in this country for more than a decade now. It's still a learning journey […] I personally think that maybe there must be some time given us to relearn what we have learnt already and then learn what we are supposed to learn.

(Asian Indian IMG female ST4+ psychiatry)

##### Lack of trust

Many IMGs felt UKG trainers did not appreciate the challenges they faced and trainers reported finding it challenging to help some IMGs—one white UKG trainer wondered whether differences were sometimes too large to be overcome. Only one trainer, a BME UKG, said more effort should be made to help IMGs adjust. Many UKGs were concerned that IMGs' prior training—especially in communication skills—did not prepare them for UK medicine, and thought IMGs may have attended medical schools with lower standards. Some UKGs felt IMGs in or coming from locum jobs were poor at communicating and/or disinterested in education; however, many IMGs found it very frustrating that locum jobs did not provide training opportunities, and several non-European Economic Area IMGs and one foreign national UKG said difficulties getting a visa or ineligibility prevented them getting jobs with good learning opportunities. A few white UKGs said BME UKGs and IMGs were more likely to be pushed into medicine.

My experience probably comes from a lot of locum doctors who are trying to get more established in the UK. I think perhaps there may be less trust from a senior perspective to somebody coming into that environment and therefore you don’t also give them the time to help support as much as you would somebody who is in a more permanent post here. […] I just feel a little bit unnerved when somebody hasn’t trained here.

(Trainer white UKG female medicine)

With time and effort trainers could bridge cultural gaps and get a better understanding of trainees' abilities. White UKGs trainers described how getting to know their IMG trainees over several months built trust and understanding and led to positive outcomes; however, trainers did not always have that time. More junior trainees moved jobs frequently, meaning relationships had to be formed quickly and trainees were under pressure to prove themselves. This was perceived to disadvantage IMGs but also BME UKGs who were less likely to ‘fit the mould’ (Asian Pakistani UKG female ST4+surgery).

##### Bias, belonging and fitting in

Reports of overt racism were rare. Subtle bias on the part of those training, assessing and recruiting trainees—even if not deliberate—was widely considered to be a cause of differential attainment, especially of the ethnic differences within UKGs.

I was with a GP a couple of weeks ago having a coffee with him. He's like, “Oh, yeah, normally when we recruit people we look at whether they’re going to mingle with us, they’re going to gel with the kind of background we are, whether they can come to barbecues with my family”. I thought to myself, “That is what my dad had to experience when he first came to this country and was rejected by society”.

(Asian Pakistani UKG female ST1–3 GP)

F1: There's still quite a lot of sponsorship that goes on. So rather than there being a meritocracy in terms of mentoring, certain trainees will sponsored as the chosen ones. And those factors that define chosen ones can be varied depending on speciality, so they could include gender, ethnicity, where you went to school. (White UKG female ST4+ medicine)

M1: Choice of sport. (White UKG male ST4+ medicine)

F1: Who you're married to.

F2: What your accent is. (White UKG female ST1–3 medicine)

F1: All sorts of things, I've seen it all, it still goes on.

Some BME UKGs remarked that it was only because they spoke with middle class accents and went to a medical school with a good reputation that they did not suffer discrimination; many IMGs felt their accent made people immediately question their ability, made them less likely to be recruited, and more likely to fail examinations. Several BME UKGs felt they had not personally suffered discrimination, although in our sample BME UKGs were more likely than white UKGs to believe that there was an ethnic attainment gap. One BME UKG described why she did not want to think she had been discriminated against:

I’m not going to start assuming [discrimination], because if you start assuming that, that's a very slippery slope. You just then think, you become very paranoid. You start thinking that everyone is out to get you. […] If you try and—this sounds really awful saying this—but if you try and blend in and just get on with everyone and, you know, you come across less problems. No one likes the one who's going to kick up a fuss or start saying “Oh, it's because I’m an ethnic minority this, that and the other”. No, you start getting yourself into problems if you start thinking like that.

(Asian other UKG female ST1–3 medicine)

Many trainers acknowledged that bias could exist but white trainers were more likely to say medicine was unbiased. A GP trainer said that he felt as a white UK male he had the fewest opportunities. In contrast, a BME trainer remarked “you are probably less likely to be successful the more different you are from the people assessing you” (trainer black UKG medicine).

#### Relationships with peers

Peers provided practical support and advice, solidarity, understanding and emotional support. Trainees tended to seek support from others within the same cultural group, even within the UKG group:

Ever since medical school I've pretty much hung around with the ethnic minority people, I don't know why actually. And then you see other groups that are all white.

(Mixed UKG female ST1–3 GP)

UKGs describe organising opportunities to get together in person or online to share knowledge and provide emotional support—something they felt IMGs missed; however, many IMGs said they particularly valued the opportunity to meet other IMGs who could be trusted to understand and not to judge, and described supporting junior IMG colleagues. A few IMGs felt integration and immersion in the UK culture was important.

#### Hidden curriculum: the culture of medicine

Medicine was perceived as a vocation that demanded hard work, long hours and personal sacrifice, and where success or failure was largely determined by individual factors such as motivation. Experiencing difficulties was seen as a sign of weakness, meaning trainees felt they were not always given the support needed to learn or were blamed for problems that were not their fault. IMGs could feel stigmatised or disadvantaged by attending extra courses. Reputations were thought to follow trainees between jobs, which made it hard to report bullying including ‘race’-related problems. This was perhaps amplified for IMGs and BME UKGs who were more likely to report seniors not believing in or trusting them.

I’ve gotten used to sometimes if I tell people I’m an ST7 in Medicine they almost seem surprised.

(Black UKG female ST4+ medicine)

Just imagine someone starting on F2 being told to stay in an Acute Care bay, which is the really deep end. […] The next morning I called the consultant, it was a professor, and I told him that I struggled overnight, and unfortunately […] the registrar was not very supportive that night, and I told him that I struggled overnight, I think I should be in a place where I could grow. […]. But unfortunately that experience was misinterpreted […] for being a weakness. […] [My educational supervisor] told me that “Oh you need to go back to become an F1”. […] I was in tears.

(Black IMG male ST1–3 medicine)

#### Fairness of assessments and recruitment

Royal College examinations were generally perceived as more rigorous and fair than Annual Review of Competence Progression (ARCP) assessments and recruitment. UKGs were more critical of ARCPs than IMGs, who were more critical of Royal College examinations. UKGs felt ARCPs could depend on good relationships with colleagues who would sign them off at the last minute and complete their multisource feedback positively, and this could be harder for IMGs and BME UKGs; however, IMGs were more likely to feel ARCPs were fair because all trainees have to tick the same boxes. Participants from all groups believed recruitment processes were vulnerable to bias and some UKG trainers had concerns about employing IMGs. IMGs described being ineligible for some training jobs.

The employers are going to look for someone who can be well integrated in their team and they might not see that in you as an ethnic minority even though it's not something that they would outright say. That's why I always say it's very subtle. They might look for something else and blame it on that: “Oh, it's because you don’t have enough experience at this or that”. Even though your CV actually might match your colleague or even be better than your colleague's.

(Black UKG female foundation)

If somebody had trained in another country and you didn’t have confidence in the registration of that qualification in that country, the people are going to be to the same standard, you might be less happy to recruit people from that environment.

(Trainer white UKG male GP)

IMG and BME UKG trainees thought communication in examinations was different from real life and described learning to “play the game” (black IMG male ST1–3 GP) to pass. Confidence was perceived as important to pass clinical examinations but IMGs were less confident because they worried their accent would disadvantage them; they knew they were statistically more likely to fail, and they knew colleagues who were good clinically who had failed. Reassurance and practical support from seniors was important to build confidence.

Trainers were more positive about ARCPs, the main criticism being that panels passed trainees they should not. Trainers felt examinations were robust and fair (many were involved in examining), even if they were harder for candidates who were unfamiliar with the UK culture and language.

#### Work–life balance

Trainees valued emotional and practical support from partners and families especially when they were having difficulties at work; but long hours, inflexible training and lack of family-friendly attitudes made it hard to get this support. Trainees lacked autonomy about where they worked and lived, especially those who did not score as highly at medical school or in recruitment tests, which is perhaps why BME UKGs and IMGs talked more frequently about ending up separated from family and the pressure this entailed.

M1: The year apart. We’ve tried a year so I deferred for a year but still couldn’t start and all my wife and kids couldn’t move up. We spent a year commuting from Sheffield to Bristol […]. (Arab UKG male ST4+ surgery)

M2: You can’t give up a [training] number, that's just a golden ticket. It's really career or family sometimes. It's tough. (White IMG male ST4+ surgery)

#### Impact of work on well-being

BME UKGs and IMGs in our sample were more likely to mention mental health problems caused by work stresses including problematic relationships with colleagues that lowered confidence, burnout, social isolation and lack of pastoral support. These problems impeded learning and performance at work.

F1: I feel, like, on constant level of burnout […] So unless I either declare myself- if I say I've actually got depression and I'm unfit to practice, then there is no way. I've been quiet before about…(Asian Indian UKG female ST1–3 psychiatry)

F2: […] I was at the point, like everyone is, when they're working where just an entire 3 months of just not sleeping at night because you're just so worried about the next day and how you're going to manage. (Asian Indian UKG female ST1–3 medicine)

I did not have any work experience, neither back home nor here. And also my Foundation training was up North and then I left my daughter and my husband here in London. […] I was really anxious during that time.[…] I could not pay attention to what was going on. […] [My educational supervisor] said “Okay, if you cannot work like this then probably you need to, you may need to think about changing your career” […] Medicine has always been my passion. I cannot think doing anything else apart from that. I got really upset.

(Asian Pakistani IMG female ST1–3 psychiatry)

#### Fear of living up to negative expectations

Many IMGs and two BME UKGs talked about the psychological pressure of knowing that they may be subject to negative stereotyping or failure, with one IMG wondering whether “we just aren't as clever as the local trainees” (Asian other IMG male ST4+ surgery).

During my training I have seen lots of local trainees or white doctors, they are not doing that much work, and then in fact the other doctors—we are immigrant doctors—they have been given more work to do, and then they still do it, but they are still considered inefficient. […] We need to work twice as much as, twice as hard as the local trainees does to be half as good as they are.

(Asian Pakistani IMG female ST1–3 psychiatry)

I’m expecting to get a lower mark because I’m- I know it's a stupid way of thinking but actually it got to the point where I was thinking “What is it? Am I…?” I wasn’t sure if it was my knowledge anymore, I wasn’t sure if it was my confidence, I wasn’t sure if it was my skin colour. So you start-I think it creates almost like a nasty way of thinking and how you perceive yourself to be. And if that someone's expectation of you is low subconsciously, your performance will be low.

(Black UKG female ST4+ psychiatry)

## Discussion

### Statement of principal findings

In this national study of trainees' experiences of postgraduate medical training, most trainees reported difficulties, but BME UKGs and IMGs faced additional difficulties that impeded learning and performance. Relationships with senior doctors were considered crucial to learning but were more problematic for BME UKGs and IMGs, which was perceived to result partly from bias. IMGs faced cultural differences and lack of trust from seniors, and many looked to IMG peers for support instead. A culture in which success is determined by drive and ability, and difficulties are a sign of weakness could make it hard to access support, and additional training for IMGs could be stigmatising. Workplace-based assessment and recruitment processes were widely considered vulnerable to bias whereas examinations were considered more rigorous. Relationships outside work were an important source of emotional support but lack of work–life balance and lack of autonomy about geographical location of work could mean separation from family, especially for BME UKGs and IMGs, several of who reported mental health problems that impacted on work.

### Strengths and weaknesses of the study

This large-scale qualitative study provides new insights into the causes of ethnic differences in attainment among UKGs, which to date have been little understood, making it difficult to develop interventions. This study points to several areas for interventions to focus on. The study is novel in exploring similarities in the causes of differential attainment within UKGs and between UKGs and IMGs, facilitating the development of interventions to address both.

Our analysis was based on factors identified in an international study of HE^24^ reflecting that differential attainment is a widespread problem. Our study aimed to understand the issues in depth rather than to provide statistical generalisations; however, its theoretical foundations allow theoretical generalisability.^28^ Trainee interviews were contextualised by trainer interviews; interviewees were purposively sampled to provide a spread across different specialties, geographic areas and stages of training; and the data were analysed by a linguist, psychologists and medics—all of which improved reliability and validity.

Poor recruitment from some specialties, for example, radiology, did not allow us to look at differences between specialties. The large number of GP trainers could have skewed the trainer findings, although we also interviewed 14 trainers from hospital medicine. With all research it is possible that participants had particular reasons for taking part. Data were collected in November and December 2015 just after junior doctors in England voted to strike over the Government's imposition of a new contract.^29^ This may have encouraged participants to speak negatively about their training, but there is little to suggest that white and BME doctors or IMGs and UKGs view the concerns surrounding junior doctor contracts differently.

### Strengths and weaknesses in relation to other studies, discussing important differences in results

The central role of the teacher–learner relationship in medical and other adult education is well known,^30^ ^31^ and it is known that teacher–learner relationships in medical students can be impeded by ethnic differences.^32^ ^33^ The perception that bias can affect learning is reflected by national surveys reporting that newly qualified BME UKGs were less likely to agree ‘the NHS is a good equal opportunities employer for doctors from ethnic minorities’^34^ and were less satisfied with their training^35^ although IMGs were more satisfied than UKGs.^35^ It may be that IMGs have different expectations—one IMG in our study expected to be discriminated against, feeling it was natural to prefer one's own (black IMG ST1–3 medicine). IMGs in our study reported worrying they were going to fail or be disadvantaged in examinations—a form of stereotype threat that impedes minority students' performance in education generally^36^ but that has been relatively understudied in medical education. The culture of long hours, hard work, lack of work–life balance and difficulties being a sign of weakness is well known^31^ but previous research has not to the best of our knowledge considered whether it may adversely affect BME or IMG doctors particularly, although lack of social support in IMG psychiatrists in the USA is associated with increased mental health problems.^37^ The finding that trainees tended to seek support among their own cultural group fits with previous medical school research.^33^ ^38^

### Meaning of the study: possible explanations and implications for clinicians and policymakers

Trainers need time to develop good relationships with trainees, which can be difficult due to clinical pressures. The widespread belief that bias could affect trainers' perceptions of trainees during training, assessments and/or recruitment does not mean that trainers were necessarily biased; however, more could be done to raise awareness of the potential of even quite subtle bias to affect minority trainees during training as well as during assessments; but care should be taken to avoid stigmatising trainees with interventions. A lack of work–life balance and autonomy over job locations could prevent trainees from benefitting from social support outside work and affected their well-being. This may be especially problematic for BME UKGs and IMGs who—because of poorer academic performance—may have even less choice, and thus be more likely to be socially isolated and suffer mental ill health, which could impact patient care. Changes to systems to increase work–life balance and autonomy, therefore, have the potential to reduce differential attainment.

### Unanswered questions and future research

Further research is needed to determine the prevalence of the problems identified within the entire population and to examine how organisational systems affect the relationships and well-being of trainees from different ethnic and cultural groups, especially because doctor well-being impacts patient care.^39^ There is increasing evidence about the fairness of Royal College examinations, but more work is needed to examine the fairness of all assessments, especially workplace-based assessments and recruitment. This research provides the basis for interventions, but these need to be developed, trialled and rigorously evaluated.
