# Supplementary material for: Perceived causes of differential attainment in UK postgraduate medical training: a national qualitative study
Source: BMJ Open. 2016 Nov 25;6(11):e013429. doi: 10.1136/bmjopen-2016-013429 (PMC5168507; doi:10.1136/bmjopen-2016-013429)
Supplement: supplementary data [file bmjopen-2016-013429supp.pdf]

Interview schedules

One-to-one interviews - trainees:

Thank you for taking part in this research, which aims to explore the experiences of doctors in training, particularly concentrating on the fairness of training in relation to a doctor's ethnicity and the country in which a doctor went to medical school.

I'd like to audio record this interview and take some notes to help me accurately remember what was said. The recording will be sent to a professional independent transcriber, and we will remove identifiable features to anonymise the interviews. All notes will also be anonymised. The data will be kept confidential and only published in a way that means it cannot be attributed to you as an individual. Is that OK?

In a moment I'm going to be asking you a series of questions. There are no right or wrong answers, I just want to hear your opinions.

[If phone interview TURN ON TAPE and ask: can you just confirm your consent to take part for the tape?]

1. Tell me a bit about yourself

i) What's your current job? (*prompt: stage of training, specialty if appropriate, deanery/LETB*)

2. I'm going to ask some questions about the experiences you've had working as a doctor in training in the UK.

i) Think of a time when you felt you really learned a lot. (*if necessary: What happened?)*

What was it about the experience that helped you learn? (*prompt: supervision from senior colleagues, other trainees involved?)*

Did the experience change you in any way? (*prompt: how motivated you felt? the direction of your career?*)

ii) Think of a time when you really didn't learn much at all. *(if necessary: What happened?)*

What was it about the experience that hindered your learning? (*prompt: supervision from senior colleagues, other trainees involved?)*

Did the experience change you in any way? (*prompt: how motivated you felt? the direction of your career?*)

iii) Now I'd like you to think about a time when something happened that was difficult to deal with. *(if necessary: What happened?)*

What was it about the experience that made it so difficult?

Did you get any support to help you deal with it? *(prompt: Did you talk to anyone about it? Who? Can you remember what they said to you about it?)*

Did the experience change you in any way?

3. Sometimes you can get two trainees in what is essentially the same job but one learns a lot and the other learns very little.

Why do you think that might be?

4. Thinking again about your own career working as a doctor in the UK

i) What are the main challenges or hurdles that you have had to deal with professionally to get to where you are today in your career? *(prompt: getting through assessments, getting through selection processes)*

> Did you get any help or support? From whom? (*prompt: How about outside of work?)*
>
> Did anyone or anything hinder you? *(prompt: opportunities provided by the workplace; peers, senior colleagues)*
>
> *If they mention more than one challenge:* Of all the challenges you've talked about, which would you say was the most challenging? If necessary: what made it so challenging?

ii) We are particularly interested in assessments, including ARCPs and Royal College examinations.

> What comes into your head when I say "ARCP"?
>
> How fair do you think ARCPs are?
>
> What about Royal College exams? How fair are they?
>
> Have you ever failed an exam or an ARCP? *(prompt: if exam, was it written or clinical)*
>
> > Why do you think you failed?
> >
> > Do you think failing affected you in any way? *(if necessary: How?)*

b) Now let's look to the future

i) Do you have an idea of where you ultimately would like to get to career-wise?

> *If yes:* Where is that?

ii) What are the main challenges or hurdles that you are going to have to deal with professionally in the next few years?

> Will you need any help or support to deal with those challenges or hurdles? What kind? From whom? *(Prompt: other trainees, senior colleagues, anyone outside work?)*
>
> How easy or difficult do you think it will be to get the help and support you need?

5. Thinking about trainees in general now

i) Evidence shows that UK-trained doctors from minority ethnic groups (in other words people who would not tick the 'white' box on an ethnic monitoring form) are less likely to be successful in recruitment and in assessments compared to UK-trained doctors who are white.

> What do you think about this?
>
> Why do you think this might be the case?
>
> What could be done to reduce this difference?

ii) Evidence also shows that doctors who trained outside of the UK are less likely to be successful in recruitment and in assessments compared to UK-trained doctors.

> What do you think about this?
>
> Why do you think this might be the case?
>
> What could be done to reduce this difference?

Focus group - trainees:

Thank you for taking part in this research, which aims to explore the experiences of doctors in training, particularly concentrating on the fairness of training in relation to a doctor's ethnicity and the country in which a doctor went to medical school.

We are going to be audio recording this focus group and my colleague [name] will be taking notes. This is to help us accurately remember what everyone says. The recording will be sent to a professional independent transcriber, and we will remove identifiable features to anonymise the interviews. All notes will also be anonymised. The data will be kept confidential and only published in a way that means it cannot be attributed to you as an individual.

We would also like you to agree to keep everything you your colleagues say in this room confidential. Can we agree that? [make sure everyone agrees].

In a moment I'm going to be asking you a series of questions. There are no right or wrong answers, we just want to hear your opinions. This focus group is not about reaching consensus, we are interested in hearing a variety of different opinions.

1. Tell me a bit about yourself

i) What's your name and your current job? (*prompt: stage of training, specialty if appropriate, deanery/LETB*) [ask everyone in turn]

2. I'm going to ask some questions about the experiences you've had working as a doctor in training in the UK.

i) Think of a time when you felt you really learned a lot. (*if necessary: What happened?)*

> What was it about the experience that helped you learn? (*prompt: supervision from senior colleagues, other trainees involved?)*
>
> Did the experience change you in any way? (*prompt: how motivated you felt? the direction of your career?*)

ii) Think of a time when you really didn't learn much at all. *(if necessary: What happened?)*

> What was it about the experience that hindered your learning? (*prompt: supervision from senior colleagues, other trainees involved?)*
>
> Did the experience change you in any way? (*prompt: how motivated you felt? the direction of your career?*)

iii) Now I'd like you to think about a time when something happened that was difficult to deal with. *(if necessary: What happened?)*

> What was it about the experience that made it so difficult?
>
> Did you get any support to help you deal with it? *(prompt: Did you talk to anyone about it? Who? Can you remember what they said to you about it?)*
>
> Did the experience change you in any way?

3. Sometimes you can get two trainees in what is essentially the same job but one learns a lot and the other learns very little.

Why do you think that might be?

4. Thinking again about your own career working as a doctor in the UK

i) What are the main challenges or hurdles that you have had to deal with professionally to get to where you are today in your career? *(prompt: getting through assessments, getting through selection processes)*

Did you get any help or support? From whom? (*prompt: How about outside of work?)*

Did anyone or anything hinder you? *(prompt: opportunities provided by the workplace; peers, senior colleagues)*

If they mention more than one challenge: Of all the challenges you've talked about, which would you say was the most challenging? (*If necessary*: what made it so challenging?)

ii) We are particularly interested in assessments, including ARCPs and Royal College examinations.

What comes into your head when I say "ARCP"?

How fair do you think ARCPs are?

What about Royal College exams? How fair are they?

Anyone failed an exam or an ARCP? *(prompt: if exam, was it written or clinical)*

Why do you think you failed?

Do you think failing affected you in any way? *(if necessary: How?)*

b) Now let's look to the future

i) Do you have an idea of where you ultimately would like to get to career-wise?

If yes: Where is that?

ii) What are the main challenges or hurdles that you are going to have to deal with professionally in the next few years?

Will you need any help or support to deal with those challenges or hurdles? What kind? From whom? *(Prompt: other trainees, senior colleagues, anyone outside work?)*

How easy or difficult do you think it will be to get the help and support you need?

5. Thinking about trainees in general now

i) Evidence shows that UK-trained doctors from minority ethnic groups (in other words people who would not tick the 'white' box on an ethnic monitoring form) are less likely to be successful in recruitment and in assessments compared to UK-trained doctors who are white.

What do you think about this?

Why do you think this might be the case?

What could be done to reduce this difference?

ii) Evidence also shows that doctors who trained outside of the UK are less likely to be successful in recruitment and in assessments compared to UK-trained doctors.

What do you think about this?

Why do you think this might be the case?

What could be done to reduce this difference?

One-to-one interview – trainer

Thank you for taking part in this research, which aims to explore the experiences of doctors in training, particularly concentrating on the fairness of training in relation to a doctor's ethnicity and the country in which a doctor went to medical school.

I'd like to audio record this interview and take some notes to help me accurately remember what was said. The recording will be sent to a professional independent transcriber, and we will remove identifiable features to anonymise the interviews. All notes will also be anonymised. The data will be kept confidential and only published in a way that means it cannot be attributed to you as an individual. Is that OK?

In a moment I'm going to be asking you a series of questions. There are no right or wrong answers, I just want to hear your opinions.

[If phone interview TURN ON TAPE and ask: can you just confirm your consent to take part for the tape?]

1. Tell me a little bit about yourself

i) Where did you go to medical school and when did you graduate?

ii) What's your clinical job (*prompt: specialty, deanery*)?

iii) What responsibilities do you have as a trainer?

2. I'd like to ask you some questions about your experiences as a trainer

i) Think of a time at work when you felt your trainees, or one trainee in particular, learned a lot *(if necessary: What happened?)*

Why was it that they learned so much, do you think?

Did that experience change the way you approach your role as a trainer in any way?

ii) Think of a time at work when you felt your trainees, or one trainee in particular, really didn't learn anything *(if necessary: What happened?)*

Why was it that the learning didn't happen, do you think?

Did that experience change the way you approach your role as a trainer in any way?

3. Sometimes you can get two trainees in what is essentially the same job but one learns a lot and the other learns very little.

Why do you think that might be?

4. What are the main challenges or hurdles that trainees have to deal with in their careers?

Of all those challenges, which would you say was the most difficult? Why?

What help or support do you think trainees need in dealing with those challenges? *(prompt: How about outside of work?)*

What do you think are the main things that hinder trainees' progression? *(Prompt: opportunities provided by the workplace)*

5. We are very interested in assessments, ARCPs and Royal College exams.

What comes into your head when I say "ARCP"?

How fair do you think ARCPs are?

What about Royal College exams? How fair are they?

6. Thinking about trainees in general now

i) Evidence shows that UK-trained doctors from minority ethnic groups (in other words people who would not tick the 'white' box on an ethnic monitoring form) are less likely to be successful in recruitment and in assessments compared to UK-trained doctors who are white.

What do you think about this?

Why do you think this might be the case?

What could be done to reduce this difference?

ii) Evidence also shows that doctors who trained outside of the UK are less likely to be successful in recruitment and in assessments compared to UK-trained doctors.

What do you think about this?

Why do you think this might be the case?

What could be done to reduce this difference?

7. Anything else you wanted to say, or that you wish I'd asked you about?

Focus group - trainers:

Thank you for taking part in this research, which aims to explore the experiences of doctors in training, particularly concentrating on the fairness of training in relation to a doctor's ethnicity and the country in which a doctor went to medical school.

We are going to be audio recording this focus group and my colleague [name] will be taking notes. This is to help us accurately remember what everyone says. The recording will be sent to a professional independent transcriber, and we will remove identifiable features to anonymise the interviews. All notes will also be anonymised. The data will be kept confidential and only published in a way that means it cannot be attributed to you as an individual.

We would also like you to agree to keep everything you your colleagues say in this room confidential. Can we agree that? [make sure everyone agrees].

In a moment I'm going to be asking you a series of questions. There are no right or wrong answers, we just want to hear your opinions. This focus group is not about reaching consensus, we are interested in hearing a variety of different opinions.

1. Tell me a little bit about yourself

i) What is your name? Where did you go to medical school and when did you graduate?

ii) What's your clinical job (*prompt: specialty, deanery*)?

iii) What responsibilities do you have as a trainer?

2. I'd like to ask you some questions about your experiences as a trainer

i) Think of a time at work when you felt your trainees, or one trainee in particular, learned a lot *(if necessary: What happened?)*

Why was it that they learned so much, do you think?

Did that experience change the way you approach your role as a trainer in any way?

ii) Think of a time at work when you felt your trainees, or one trainee in particular, really didn't learn anything *(if necessary: What happened?)*

Why was it that the learning didn't happen, do you think?

Did that experience change the way you approach your role as a trainer in any way?

3. Sometimes you can get two trainees in what is essentially the same job but one learns a lot and the other learns very little.

Why do you think that might be?

4. What are the main challenges or hurdles that trainees have to deal with in their careers?

Of all those challenges, which would you say was the most difficult? Why?

What help or support do you think trainees need in dealing with those challenges? *(prompt: How about outside of work?)*

What do you think are the main things that hinder trainees' progression? *(Prompt: opportunities provided by the workplace)*

5. We are very interested in assessments, ARCPs and Royal College exams.

What comes into your head when I say "ARCP"?

How fair do you think ARCPs are?

What about Royal College exams? How fair are they?

6. Thinking about trainees in general now

i) Evidence shows that UK-trained doctors from minority ethnic groups (in other words people who would not tick the 'white' box on an ethnic monitoring form) are less likely to be successful in recruitment and in assessments compared to UK-trained doctors who are white.

What do you think about this?

Why do you think this might be the case?

What could be done to reduce this difference?

ii) Evidence also shows that doctors who trained outside of the UK are less likely to be successful in recruitment and in assessments compared to UK-trained doctors.

What do you think about this?

Why do you think this might be the case?

What could be done to reduce this difference?

7. Anything else you wanted to say, or that you wish I'd asked you about?
